# Human immunodeficiency virus infection and autoimmune hepatitis during highly active anti-retroviral treatment: a case report and review of the literature

**DOI:** 10.1186/1752-1947-5-233

**Published:** 2011-06-25

**Authors:** Hanady Daas, Riad Khatib, Haitham Nasser, Farah Kamran, Martha Higgins, Louis Saravolatz

**Affiliations:** 1 Department of Internal Medicine, Division of Infectious Diseases, St John Hospital & Medical Center, 19251 Mack Avenue, Suite 340, Grosse Pointe Woods, MI 48236, USA; 2 Department of Pathology, St John Hospital & Medical Center, 22101 Moross Road, Detroit, MI 48236, USA; 3 Department of Internal Medicine, St John Hospital & Medical Center, 22101 Moross Road, Detroit, MI 48236, USA

## Abstract

**Introduction:**

The emergence of hepatic injury in patients with human immunodeficiency virus infection during highly active therapy presents a diagnostic dilemma. It may represent treatment side effects or autoimmune disorders, such as autoimmune hepatitis, emerging during immune restoration.

**Case presentation:**

We present the case of a 42-year-old African-American woman with human immunodeficiency virus infection who presented to our emergency department with severe abdominal pain and was found to have autoimmune hepatitis. A review of the literature revealed 12 reported cases of autoimmune hepatitis in adults with human immunodeficiency virus infection, only three of whom were diagnosed after highly active anti-retroviral treatment was initiated. All four cases (including our patient) were women, and one had a history of other autoimmune disorders. In our patient (the one patient case we are reporting), a liver biopsy revealed interface hepatitis, necrosis with lymphocytes and plasma cell infiltrates and variable degrees of fibrosis. All four cases required treatment with corticosteroids and/or other immune modulating agents and responded well.

**Conclusion:**

Our review suggests that autoimmune hepatitis is a rare disorder which usually develops in women about six to eight months after commencing highly active anti-retroviral treatment during the recovery of CD4 lymphocytes. It represents either re-emergence of a pre-existing condition that was unrecognized or a *de novo *manifestation during immune reconstitution.

## Introduction

Impaired immunity in individuals with human immunodeficiency virus (HIV) infection affects the defense mechanisms against pathogens and alters the regulation of autoimmunity [1]. This may lead to the emergence of autoimmune disorders or modification of pre-existing conditions. Several conditions may remit, such as systemic lupus erythematosus (SLE), while others, such as psoriasis, intensify. The development of liver disease during highly active anti-retroviral treatment (HAART) in patients with HIV infection without evidence of co-infection with hepatitis viruses poses a diagnostic dilemma. This may be due to treatment side effects or to the emergence of autoimmune disorders during immune restoration.

Autoimmune hepatitis (AIH) is rare in patients with HIV infection. Additionally, hepatic involvement is unusual in other common autoimmune disorders. We present the case of a patient with AIH and SLE emerging *de novo *during HAART and review all previously reported cases of AIH in patients with HIV infection who are undergoing HAART.

## Case presentation

A 42-year-old African-American woman who had been diagnosed with HIV infection in 1989 acquired by heterosexual contact had a fluctuating CD4 count and a viral load secondary to non-adherence. In March 2009, she was extensively counseled on adherence to treatment and was started on a new regimen that included emtricitabine/tenofovir and etravirine. She became more compliant with treatment, and her clinical parameters improved. Before March 2009, her CD4 had been 157 cells/mm^3 ^and her viral load had been 120,000 copies/mL. One month after treatment adjustment, her CD4 went up to 232 cells/mm^3 ^and her viral load was undetectable. There was no personal or family history of autoimmune disease.

Six months after treatment adjustment she started to experience gradual right upper quadrant pain associated with intermittent night sweats. Her pain increased in intensity and became intractable. A computed tomographic scan of her abdomen was unremarkable. She was seen in the office with fever and tachycardia and was hospitalized because of possible sepsis and acute abdomen.

Her physical examination revealed that she was febrile (body temperature 102.1°F), tachycardic (130 beats/min) and hypoxic (O^2 ^saturation 84% on room air). Her chest examination revealed fine bibasilar crackles. Her abdominal examination demonstrated diffuse abdominal tenderness with rebound that was most prominent in the right upper quadrant.

A hepatobiliary iminodiacetic acid scan showed patent biliary ducts with a normal gallbladder ejection fraction. Computed tomography of the chest showed pericardial effusion that was confirmed by a transthoracic echocardiogram.

**Figure 1:**
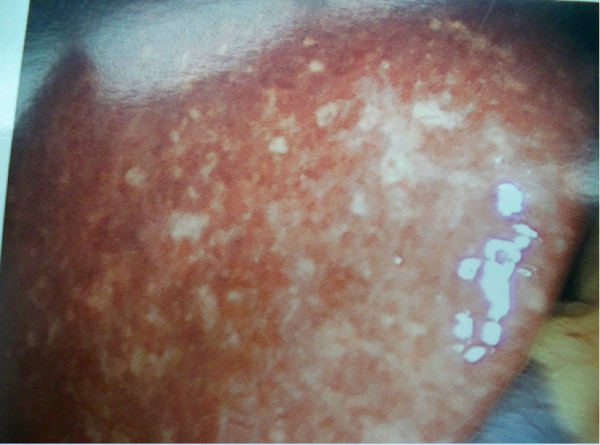
**Laparoscopic image of the liver showing diffuse whitish-gray plaque without nodularity or cirrhosis**.

**Figure 2:**
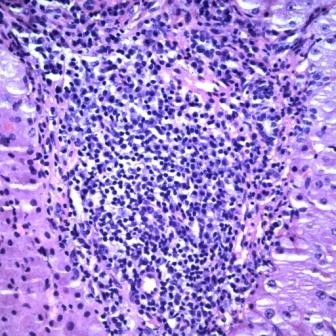
**Liver biopsy (hematoxylin and eosin stain) showing dense portal lymphoplasmacytic infiltrate and centrilobular hepatocellular necrosis caused by an acute chronic inflammatory infiltrate suggestive of autoimmune hepatitis**.

On day 3 of her hospitalization, she underwent a pericardial window, a pericardial biopsy and a laparoscopy with liver biopsy. The laparoscopy revealed a grossly abnormal liver (Figure 1). The liver biopsy demonstrated a dense portal lymphoplasmacytic infiltrate with multifocal zones of hepatocellular centrilobular necrosis consistent with AIH (Figure 2). Histological staining for fungi and mycobacterium were negative.

Pertinent laboratory findings in this patient included alanine aminotransferase 1526 U/L, aspartate aminotransferase 777 U/L, international normalized ratio, 1.53; albumin level, 2.7 g/dL; anti-nuclear antibody (ANA) titer, 1:1280; negative anti-smooth muscle antibody; negative anti-cardiolipin and anti-ribosomal antibodies; anti-double-stranded DNA (anti-dsDNA) titer, 1:160; and immunoglobulin G level, 4600 mg/dL. Her antibodies to hepatitis viruses A, B and C and hepatitis B surface antigen were negative.

Given her clinical picture, her positive laboratory test for ANA and anti-dsDNA and the histopathology of her liver biopsy, a diagnosis of SLE with AIH was made. Her calculated AIH score was 19 (> 15 is considered a definite diagnosis according to the International Autoimmune Hepatitis Group criteria).

The patient was initiated on high-dose steroid therapy (40 mg every 12 hours). By the next day, her abdominal pain had improved, and she was discharged from the hospital on a tapering dose of steroids.

One year after her hospitalization the patient remained in remission, with normal liver function and suppressed HIV viral load. Her steroid therapy was tapered off and stopped completely two months after being discharged from the hospital.

## Discussion

Our review of the literature revealed 12 cases of autoimmune hepatitis in patients with HIV infection [2-8]. Three had co-infections with hepatitis C virus and were receiving interferon therapy [6-8] and six more had AIH before starting HAART and one pediatric patient's data were missing. Only three patients developed AIH after starting HAART, similar to our patient. The clinical characteristics of the reviewed cases and our case are shown in Table 1. All patients who received HAART prior to AIH had a significant rise in CD4 count and undetectable HIV RNA before AIH was diagnosed. Two patients had other concomitant autoimmune diseases, one with Grave's disease and the other with diffuse infiltrative lymphocytic syndrome. This review illustrates that AIH in patients with HIV infection on HAART is rare. It has been encountered in women who had significant elevations in CD4 count, suggesting the emergence of AIH during immune restoration. It presented insidiously with non-specific manifestations. The diagnosis is usually based on AIH score, the absence of other conditions and characteristic histopathological findings.

**Table 1:** Characteristics of 11 reported patients with concomitant HIV and AIH^a^

Case reports	Patient age (years)/gender	Onset	CD4^b^/CD4^c^	VL^b^/VL^c^	AIH score	Outcome	Other AI diseases	ART
German *et al. *[2]	38^d^/man	Chronic	216/384	81,000/< 50	Probable	Excellent	Vetilligo	Yes
Coriat and Podevin [7]	48/woman	Acute	250	Undetectable	Probable	Died	None	Yes
Puius *et al. *[3]	29/man	Chronic	259/174	7122/27,732	Probable	Excellent	None	Yes
	45^d^/woman	Chronic	253/297	8687/< 50	Probable	Excellent	DILS	Yes
	65/woman	Acute	200/922	Undetectable/< 75	Definite	Excellent	None	Yes
O'Leary *et al. *[4]	44^d^/woman	Chronic	269/526	4927/< 50	Definite	Excellent	Grave's disease	Yes
Wan *et al. *[5]	56/man	Chronic	331/NA	232,734/NA	Probable	Died	None	
	54/man	Chronic	357/213	5104/NA	Probable	Probable	Cirrhosis	Yes
	55/woman	Chronic	174/NA	< 50/NA	Probable	Probable	Poor	Yes
	49/woman	Acute	286/NA	69,318/NA	Definite	Definite	Died	Yes
Our patient	42^d^/woman	Acute	157/232	120,000/< 50	Definite	Excellent	SLE	Yes

^a^Two patients are not included because of lack of complete clinical data. HIV = human immunodeficiency virus; AIH = autoimmune hepatitis; AI, autoimmune; ART = antiretroviral treatment; VL = viral load; DILS = diffuse infiltrative lymphocytic syndrome; SLE = systemic lupus erythematosus; ^b^prior to initiating highly active antiretroviral treatment (HAART); ^c^after initiating HAART; ^d^patients with significant viral suppression on HAART who developed AIH.

In patients who develop liver function abnormalities while receiving HAART, it is important to exclude drug-induced liver disease. In our patient, the pathognomonic findings on the liver biopsy and the fact that she had been taking these medications long before she developed symptoms indicate that a drug reaction was not likely.

The prognosis associated with AIH in patients with HIV infection appears to be variable based on a review of 11 out of the 12 reported cases (one reported case's data were missing) of AIH in patients with HIV infection. Two patients died while receiving interferon therapy for hepatitis C virus that triggered fulminant AIH, and one died as a result of severe *Pneumocystis jiroveci *pneumonia while receiving high-dose steroids for the treatment of AIH.

Our patient had evidence of SLE in addition to AIH. Whether AIH is a manifestation of SLE or is unrelated is unclear. Liver involvement associated with AIH is relatively rare, ranging from 1.2% to 2% in patients with SLE who do not have HIV infection [9]. To date, it has not been reported simultaneously with SLE in patients with HIV infection.

The precise mechanism causing the emergence or unmasking of autoimmune conditions in patients who are HIV-positive who commence anti-retroviral therapy is complex and involves multiple cytokines and lymphocyte subsets [10-14]. Th17 cells have recently been implicated in association with chronic autoimmunity phenomena and especially in patients with HIV infection and primates with simian immunodeficiency virus [10]. An additional subset of CD4^+ ^regulatory T cells (Treg) has been described. It constitutively expresses CD25 and the transcription factor FoxP3 and has regulatory functions [1]. Although the levels of Treg in patients who are HIV-positive with autoimmune manifestations have not been reported, it seems plausible to propose that preferential depletion of Treg in some individuals could account for the increased autoimmune phenomena in some patients with acquired immunodeficiency syndrome [15-18].

## Conclusion

The present case report and review of the literature describes a rare complication of immune restoration in patients with HIV infection in the era of HAART. Recognizing AIH in the context of immune reconstitution and initiating appropriate therapy can be lifesaving. Treatment of these patients appears to be similar to that of patients without HIV infection.

## Abbreviations

AIH: autoimmune hepatitis; HAART: highly active anti-retroviral therapy; HIV: human immunodeficiency virus; VL: viral load.

## Consent

Written informed consent was obtained from the patient for publication of this case report and any accompanying images. A copy of the written consent is available for review by the Editor-in-Chief of this journal.

## Competing interests

The authors declare that they have no competing interests.

## Authors' contributions

HD wrote the manuscript, collected the images and obtained consent from the patient to publish this case report. RK reviewed the literature and edited the manuscript. FK coordinated care while this patient was hospitalized. LS provided inpatient care for the patient and edited the manuscript. HN reviewed and provided legends for the pathological images. MH reviewed the pathological specimens and made the initial diagnosis. All authors read and approved the final manuscript.
